# Cross-Cultural Adaptation, Reliability, and Validity of the Greek Version of the Fremantle Neck Awareness Questionnaire (FreNAQ-GR) in Patients with Chronic Neck Pain

**DOI:** 10.3390/healthcare12191985

**Published:** 2024-10-05

**Authors:** George A. Koumantakis, Faidra Nikolaki, Foteini Kefalaki, Petros I. Tatsios, Eleftherios Paraskevopoulos, Sotiria Vrouva

**Affiliations:** 1 Laboratory of Advanced Physiotherapy, Physiotherapy Department, School of Health & Care Sciences, University of West Attica (UNIWA), 12243 Athens, Greece; phys18683069@uniwa.gr (F.N.); phys18683085@uniwa.gr (F.K.); ptatsios@uniwa.gr (P.I.T.); elparaskevop@uniwa.gr (E.P.); 2 Department of Physical Therapy, 401 Army General Hospital of Athens, 11525 Athens, Greece; svrouva@uniwa.gr

**Keywords:** chronic neck pain, assessment, self-perception, body awareness, questionnaires, cross-cultural adaptation, reproducibility, validation, psychometric properties

## Abstract

Background: Neck self-awareness, related to sensorimotor dysfunction, can be monitored with the Fremantle Neck Awareness Questionnaire (FreNAQ). The cross-cultural adaptation of the FreNAQ in Greek (FreNAQ-GR) and an assessment of its psychometric properties were conducted. Methods: This study included 104 participants (65 female) with non-specific chronic neck pain (NSCNP). Once the cross-cultural adaptation process of the FreNAQ-GR was complete, the testing of its construct validity was conducted via an exploratory factor analysis (EFA). The construct validity examination also included a correlational analysis with a Pain Intensity Visual Analogue Scale (PI-VAS), the Neck Disability Index (NDI), the Tampa Scale of Kinesiophobia (TSK), the Pain Catastrophizing Scale (PCS), and demographics. The internal consistency of the FreNAQ-GR was also examined. A sub-sample of participants (n = 30) completed the FreNAQ-GR again after 5–7 days. Results: The dataset was appropriate for EFA (measure of sampling adequacy KMO = 0.763 and Bartlett’s test of sphericity *p* < 0.001). The FreNAQ-GR demonstrated a single-factor 6-item structure (items 7–9 removed), explaining 53.69% of the common variance. Statistically significant correlations (Spearman’s) were registered between the FreNAQ-GR (both versions) and the NDI (r = 0.33/0.29, *p* < 0.001), the TSK (r = 0.46/0.41, *p* < 0.001), and the PCS (r = 0.37/0.33, *p* < 0.001). For the 9-item and the 6-item FreNAQ-GR, the internal consistency (Chronbach’s a/McDonald’s ω) was 0.80/0.79 and 0.826/0.816, respectively. The test–retest reliability was excellent for both versions ICC_2,1_ (95% CI) = 0.98/0.98 (0.97–0.99/0.95–0.99), with low error values SEM = 0.90/0.74 and MDC_95%_ = 2.49/2.05 points. Conclusions: The FreNAQ-GR is suitable for assessing neck self-awareness in Greek-speaking patients with NSCNP.

## 1. Introduction

Neck pain (NP) is among the most common causes of pain-related disability worldwide, significantly affecting the psychosocial status of patients as well as their families [1]. Recent global burden of disease data from 204 countries show that neck pain affected 203 million people in 2020, primarily between the ages of 45 and 74, with an estimated projection of sufferers rising to 269 million by 2050 [2]. Therefore, prevention, monitoring, and reforms in the healthcare system are required to reduce the burden worldwide [1,2].

Non-specific chronic neck pain (NSCNP), in particular, is defined as pain lasting for more than 3 months that is not related to an underlying secondary pathology, clustered under the category of chronic primary pain [3]. Nonetheless, NSCNP is still difficult to manage effectively due to the complexity of its pathology, with physical [4,5] as well as psychosocial [5] factors affecting patients’ symptomatology, as well as their quality of life [6,7]. Therefore, a multifactorial assessment is required, leading to a more comprehensive understanding of the nature and extent of factors involved [8,9].

The altered somatosensory, cognitive and motor functional connectivity and processing in patients of non-traumatic NSCNP compared to healthy controls contributes to the non-resolution of NSCNP in many patients [10,11,12]. For the somatosensory domain, manifestations can include impaired endogenous pain inhibition [5] and mechanical hyperalgesia at remote non-painful sites [13]. Manifestations for the somatosensory and cognitive domains combined can include self-perceived medium to high muscular tension [4], proprioception deficits [14], and a distorted body image [9,15]. Cognitive domain disturbances include increased anxiety, depression [16], and fear-avoidance [17]. Furthermore, various motor disturbances were reported in this patient group [18,19]. As far as body image is concerned, the dysfunction present may be due to sensory-cognitive distortions in the representation of the painful body parts’ actual shape, size or sense of ‘ownership’, in the primary sensory, the pre-motor cortex [20,21], and the posterior parietal cortex [22,23]. Therefore, exercises designed to improve body awareness may be responsible for the normalization of these distortions in neural and cognitive representations as well as pain amelioration [24,25,26,27,28].

A questionnaire designed to comprehensively assess bodily self-awareness alterations in patients with chronic back pain is the Fremantle Back Awareness Questionnaire (FreBAQ) [29], upon which the Fremantle Neck Awareness Questionnaire (FreNAQ) was subsequently developed to assess the same construct in patients with NSCNP [30]. The aim of this study was to cross-culturally adapt the Fremantle Neck Awareness Questionnaire (FreNAQ) in Greek and to test its psychometric properties.

## 2. Materials and Methods

### 2.1. Sample Selection

For this study, 104 adults between 18 and 80 years old, diagnosed with NSCNP (unilateral or bilateral), of more than 3 months duration, were recruited. Participants had to be referred for physiotherapy by an orthopedic doctor and able to speak and understand Greek to be included in this study. Participants with neurological or psychological pathologies, hearing or visual impairment, vestibular system pathology, current pregnancy, previous spinal surgery, serious spinal pathology (cancer, fracture, inflammatory arthropathy), or if unwilling to consent to this study, were excluded. The data for this study were collected prior to their treatment initiation.

### 2.2. Ethics

The Ethics Committee of the University of West Attica, Athens, Greece (approval no: 53351/07.06.2022) approved the protocol of this study, according to the principles of the Declaration of Helsinki. An information sheet with the details of the aims and purposes of this study was administered to all potential participants, and those who agreed to participate completed and signed a consent form prior to their inclusion in this study.

### 2.3. Study Design

The cross-cultural adaptation process of the FreNAQ scale in Greek and validation of the scale occurred in the target language.

### 2.4. Cross-Cultural Adaptation Procedure of the FreNAQ

The cross-cultural adaptation of the FreNAQ scale in Greek was initiated with permission from Prof. Benedict Martin Wand, the scale’s developer. A previous systematically outlined methodology served as the basis for the cross-cultural adaption [31,32]. In particular, two bilingual translators (one with clinical experience in healthcare and one without) independently performed two “forward” translations from English to Greek. A draft Greek version of the scale was then produced by combining the two translations into one and discussing and resolving any minor differences. Then, without access to the original scale, two Greek-speaking language experts—one a healthcare professional and the other a non-healthcare professional—whose mother tongue was English, carried out the “back translation” of the preliminary Greek version into English. Once the back-translation process was complete, the review committee, comprising the primary researcher, a methodologist, and all of the translators, assessed each version of the FreNAQ in Greek and English to make sure that the format, language, grammar, and meaning (semantic, idiomatic, experiential, and conceptual equivalence) were appropriate. Committee members then discussed and settled upon by agreement any discrepancies, resulting in the preliminary version of the FreNAQ-GR. To verify the conceptual and semantic equivalence of the pre-final FreNAQ-GR, ten patients suffering from NSCNP participated in its initial pilot testing. On a binary scale (clear/unclear), the patients, as well as the committee members, were asked to score the questionnaire’s instructions and items. If any were unclear, participants were asked to rephrase them to make them clearer. If at least 20% of the pilot testing participants felt that any part of it was confusing, the questionnaire had to be re-evaluated and re-phrased [32].

### 2.5. Validation Procedures of the FreNAQ-GR

In the second part of this study, the finalized version of the FreNAQ-GR was administered to all participants with NSCNP referred to a private physiotherapy practice in Athens, after the consent of the practice owner was obtained.

A questionnaire including patient demographic details (age, sex, height, weight, BMI, marital status, work status, educational level, and exercise level), was administered to patients. The data collection also included the FreNAQ-GR, a Pain Intensity Visual Analogue Scale, over the past week on average (PI-VAS), the Neck Disability Index in Greek (NDI-GR), the Tampa Scale of Kinesiophobia in Greek (TSK-GR), and the Pain Catastrophizing Scale in Greek (PCS-GR). About a third of the participants (n = 30) filled in the FreNAQ-GR for a second time after 5–7 days without receiving any therapy between the two occasions for a test–retest reliability evaluation of the scale. All scales were administered to patients in printed format by either of the two student physical therapists at the time (F.N. and F.K.).

### 2.6. The Fremantle Neck Awareness Questionnaire (FreNAQ)

The FreNAQ was first cross-culturally adapted and studied for its psychometric properties in a Turkish (FreNAQ-T) [30] population and subsequently in a Japanese (FreNAQ-J) [33] population. It is a relatively new and easy to complete 9-item tool for assessing bodily self-awareness/self-perception of the neck. It is a modification of the Fremantle Back Awareness Questionnaire (FreBAQ), aimed to assess body awareness specifically for the lumbar region in individuals with chronic low back pain [29]. The FreNAQ assesses the degree of disturbed self-perception/self-awareness, such as symptoms of neglect/motor control deficits (items 1–3), reduced proprioceptive acuity (items 4–5), and changes related to the self-perception of the shape, size, and symmetrical position of the neck (items 6–9). Each item is scored using a 5-point Likert scale, with scores ranging from 0 (never) to 4 (always). The total score of the scale ranges from 0 to 36, with higher scores indicating greater levels of disturbed neck awareness [29,30,33]. Both previous studies on the FreNAQ reported the scale to be unidimensional, without including any inconsistent items (Rasch analysis), having good test–retest reliability and internal consistency [30,33]. The FreNAQ-T demonstrated significant significant, however weak correlations with the PI-VAS (during activity, at rest and at night), the SF-36 (physical fitness, physical health, vitality, pain, social functioning, general health), the Beck Anxiety Inventory, and a moderate significant correlation with the NDI [30]. Similarly, the FreNAQ-J demonstrated significant, yet weak correlations with the PI-VAS (during motion and to a lesser degree with the one at rest), the NDI, the TSK and a significant moderate correlation with the PCS [33].

### 2.7. Pain Intensity Visual Analog Scale (PI-VAS)

The pain intensity visual analog scale (PI-VAS) is a valid and reliable outcome of measuring pain severity in various pain conditions and for different timeframes. The patient must mark the level of their pain on a horizontal line 100 mm long, with the far left side of the line reading ‘no pain at all’ and the far right reading ‘worst imaginable pain’. The distance from ‘no pain at all’ to the patients’ mark indicates their pain severity. We chose the timeframe of pain ‘over the past week’ for this study, also adding the phrase ‘on average’ [34].

### 2.8. Neck Disability Index (NDI)

The Neck Disability Index (NDI) is one of the most frequently used scales to document disability in patients with neck pain [35]. The scale was constructed in 1991 and was based on the Oswestry Low Back Pain Disability Index (ODI) [36], having been translated in many languages and used in clinical and research settings [35]. The NDI possesses good to excellent psychometric properties [37]. The scale consists of 10 items inquiring about the degree that certain activities of daily living are affected by neck pain, rated from 0 to 5, the maximum score of the scale denoting maximum disability, being 50. The total score can be multiplied by two, to transform it to a percentage score [35]. The NDI was cross-culturally adapted in Greek, with very good reliability, validity, and responsiveness reported [38].

### 2.9. Tampa Scale of Kinesiophobia (TSK)

Kinesiophobia is a concept that describes the excessive, unjustified, and worsening fear that a patient feels about experiencing pain during movement [39]. Chronic pain has been found to be closely related to kinesiophobia, leading patients in a vicious cycle that perpetuates pain and functional disability [40]. The original 17-item Tampa Scale of Kinesiophobia (TSK) was used in this study, rating each item on a 4-level Likert scale, ranging from 1 (completely disagree) to 4 (completely agree) [39,41]. The total score ranges between 17 (no kinesiophobia) and 68 (maximum kinesiophobia) [41]. The TSK possesses good-excellent reliability and validity [42]. The TSK was cross-culturally adapted in patients with spinal pain, with very good reliability and validity reported [43].

### 2.10. Pain Catastrophizing Scale (PCS)

The Pain Catastrophizing Scale (PCS) measures exaggerated and negative orientations towards noxious stimuli, leading to negative thoughts relative to the pain experience [44]. The scale is made up of 13 items, each scored between 0 (never) to 4 (always), with a total score between 0 and 52, the higher the score indicating a higher perception of pain catastrophizing [44]. The scale was cross culturally adapted and validated the Greek language in patients with spinal pain [45] and in patients with chronic neck pain [46], possessing good-excellent reliability and validity.

### 2.11. Statistical Analysis

A statistical analysis was performed with the IBM Statistical Package for the Social Sciences (IBM SPSS Statistics, v.29.02.0). The distribution of continuous variables was analyzed with the Kolmogorov–Smirnov test. All descriptive statistics, as well as scores in the FreNAQ-GR, PI-VAS, NDI-GR, TSK-GR, and PCS-GR of participating patients with NSCNP were presented, depending on the distribution of each variable. If most continuous variables were not normally distributed, both the mean (SD), maximum, minimum, and the median and interquartile range (IQR) statistics were presented for all.

To assess the construct validity of the FreSHAQ-GR, we conducted an examination of its factor structure via an exploratory factor analysis (EFA) [47] and by testing for associations of the FreNAQ-GR with other relevant patient-reported outcomes [32]. The aim of the factor analysis is to achieve parsimony, by explaining the maximum amount of common variance in a correlation matrix using the minimum of explanatory factors [47]. The minimum acceptable required sample size for appropriately conducting an EFA is typically 10 participants per item [47]. To grant a stable solution, the sample should have included a minimum of 100 individuals, communalities no less than 0.40, no factor loading less than 0.32, no item cross-loadings at a level more than 0.32, and factors consisting of at least 3–5 items with strong loadings (>0.70) on one factor only [48,49]. This study adhered to the aforementioned recommendations. The items should explain at least 50% of the overall scale variance for the model to be considered sound [49]. EFA was assessed by using the principal axis factoring (PAF) method and additionally applying the Direct Oblimin rotation with the Kaiser normalization method [47]. The Bartlett test of sphericity and the Kaiser–Meyer–Olkin (KMO) measure of sampling adequacy were used to examine the sufficiency of the population used as a sample for this study. The number of factors to be extracted was determined using the Kaiser criterion (eigenvalues > 1) and by examination of the scree plot [47].

For an additional examination of the construct validity of the FreNAQ, correlations with relevant patient-reported outcomes collected (PI-VAS, NDI-GR, TSK-GR, and PCS-GR) were assessed [50]. Correlations were interpreted as negligible (0.0–0.10), weak (0.1–0.39), moderate (0.4–0.69), strong (0.70–0.89), or very strong (0.90–1.00) [51]. The required sample size to achieve 90% power with a relatively weak correlation level of r = 0.35, would be n = 82 participants, as calculated with an online program for computing sample size for correlational designs (https://sample-size.net/correlation-sample-size/ (accessed on 21 January 2023)) [52]. The significance level for all comparisons was set at 0.05. The known-group validity for participants of different sexes (Mann–Whitney U-test) and self-reported level of physical activity (Kruskal–Wallis test) was examined.

Internal consistency reliability assesses whether a set of items of a questionnaire measure the same characteristic [50]. Internal consistency was measured with the Cronbach’s α and the McDonald’s ω coefficients. The latter is recommended as an alternative to Chronbach’s α in smaller samples or when items are skewed [53]. Values above 0.70 for both internal consistency reliability coefficients are considered acceptable, above 0.80 good, and above 0.90 excellent [50]. The between-day test–retest reliability was calculated using the two-way mixed effects absolute agreement single measurement intraclass correlation coefficient (ICC_2,1_) [46] [50,54], the standard error of the measurement (SEM), and the minimum detectable change (MDC_95%_) [55]. ICCs less than 0.5 were considered poor, 0.5–0.75 moderate, 0.75–0.90 good, and greater than 0.90 excellent [54]. The SEM and MDC_95%_ indicate the error level of the measurement in the same values of the original measurement [50]. The MDC specifically represents the smallest detectable amount of change not due to measurement error [55].

Floor/ceiling effects were considered present if more than 15% of participants had a total FreNAQ-GR score of either 0 or 36 [56].

## 3. Results

### 3.1. Cross-Cultural Adaptation of the FreNAQ-GR

No special issues were encountered during the cross-cultural adaptation process of the FreNAQ, except for questions 7 and 8, where it was challenging to accurately and clearly capture the phrases “larger than it appears” and “smaller than it appears” in the Greek language, similar to a previous study of the cross-cultural adaptation of the FreSHAQ-GR [57], as well as the words in questions 7 (swollen), 8 (shrunk), and 9 (lopsided). These issues were appropriately resolved by consensus. The patients and members of the experts’ committee rated all items as ‘clear’ during the pre-final version’s pilot testing, and the subsequent validation process used the final version of the FreNAQ-GR (Supplementary Materials).

### 3.2. Descriptive Statistics

In total, 104 participants (65 women and 39 men) were included, all of working age (apart from one 70-year old). Their pain duration exceeded for all the 3-month timepoint, with a group mean pain duration period of 17.4 months. Their level of self-reported physical activity was low (n = 42) or medium (n = 42) for most of the patients, with some declaring a high level (n = 20).

The examination of normality of distribution with the Kolmogorov–Smirnov test revealed that most continuous variables (height, pain duration, FreNAQ-GR, PI-VAS, NDI-GR, and PCS-GR) were non-normally distributed; therefore, parametric and non-parametric descriptive statistics were used to present the patient demographic characteristics (Table 1) and questionnaire data (Table 2 and Table 3). Consequently, all correlational analyses were performed with Spearman’s rank-order correlation test.

No floor or ceiling effects were observed for the FreNAQ total score, with none of the participants scoring either a total of 0 or 36 (Table 2). The response frequencies per item of the FreNAQ-GR for a 0 score (“never feeling like that”) ranged between 6.7% (item 5) to 20.2% (item 2); however, slightly more than half of the participants (52.9%) selected this score for item 8. On the other end, the response frequencies per item of the FreNAQ-GR for a 4 score (“always feeling like that”) ranged between 1.9% (items 2 and 3) to 13.5% (item 9). Only a few participants (3.0%) selected scored 4 and no participants scored 3 (“often feeling like that”) for item 8 (Table 3).

### 3.3. Construct Validity of the FreNAQ-GR

**Figure 1:**
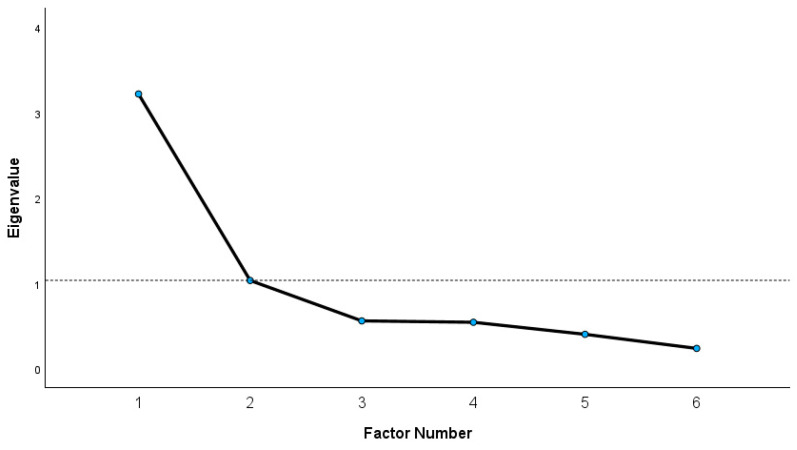
Scree plot of the eigenvalues of factors.

The initial solutions resulted with items 8, 7, and 9 having very low communalities (0.12, 0.30, and 0.25 respectively); therefore, these items were removed and the analysis was repeated with the remaining items of the FreNAQ-GR [48]. For the 6-item solution, the data collected were suitable for factor analysis, according to Bartlett’s test of sphericity, which was highly significant (chi-square = 233.60, df = 15, *p* < 0.001), and the value of the Kaiser–Meyer–Olkin measure of sampling adequacy was high (0.763) [47]. The PAF method revealed a single-factor solution with an eigenvalue of 3.22, accounting for 53.69% of the total variance. The communalities of the six items ranged from 0.40 to 0.51. The factor loadings of the items ranged from 0.63 to 0.71 (Table 4). The scree plot confirming the single factor solution is also presented (Figure 1).

Construct validity assessed via correlations of the 9-item and the 6-item FreNAQ-GR with other patient demographic characteristics and patient-reported outcomes demonstrated highly statistically significant correlations (Spearman’s index); however, the correlations were weak to moderate, respectively, with the NDI (r = 0.33/0.29, *p* < 0.001), the TSK-GR (r = 0.46/0.41, *p* < 0.001), and the PCS-GR (r = 0.37/0.33, *p* < 0.001). The correlation between the 9-item and the 6-item FreNAQ-GR was strong and highly significant (r = 0.94, *p* < 0.001), (Table 5). No significant correlations were demonstrated either with the PI-VAS over the past week on average, the pain duration, and the demographic characteristics. No differences in the FreNAQ-GR scores were identified between male-female participants or participants with a different self-reported level of physical activity.

### 3.4. Reliability of the FreNAQ-GR

For an internal consistency measurement, the 9-item FreNAQ-GR Cronbach’s α coefficient was 0.80 and the McDonald’s ω was 0.79, while the respective values for the 6-item FreNAQ-GR were 0.826 and 0.816, slightly higher than the 9-item ones. Both coefficients were considered good for the 6-item scale [50].

The test–retest between-day relative reliability measured with the ICC_2,1_ (95% CI) values was excellent (>0.90), and the indices quantifying the amount of test–retest error (SEM and MDC_95%_), representing absolute reliability, were low (Table 6) [32]. Furthermore, the MDC_95%_ value, relative to the range of values of the questionnaire (0–36), if interpreted as per cent error in relation to a grand mean of 16.32 and 10.67, respectively, for the 9- and 6-itemFreNAQ-GR, amounted to (2.49/16.32) × 100 = 15.25% and (2.05/10.67) × 100 = 19.21%.

## 4. Discussion

An impaired body awareness contributes to altered sensorimotor processing in patients with CNP [11,12], and it is contributing to the condition’s chronicity [15,23]. In addition, body awareness therapy is considered an important element in the multidisciplinary management of patients with chronic pain [24,28]. Therefore, the cross-cultural adaptation and validation [31,32] of a comprehensive, body-specific scale assessing body awareness in patients with CNP, such as the FreNAQ [30,33] in the Greek language, was deemed essential.

Patients with CNP with symptom duration of more than three months who were referred to a private practice for physical therapy were recruited to this study after fulfilling certain inclusion criteria. The sample size of the current study (n = 104) was comparable to the two previous ones on the FreNAQ-T (n = 111) [30] and the FreNAQ-J (n = 100) versions [33]. The participants’ mean age was similar to that of the previous two investigations, and the distribution of sexes was similar for this study and the FreNAQ-J study (65% female), but it was different for the FreNAQ-T study (77.4% female). The average pain duration was much less in the participants of the current study (mean ± SD of 17.4 ± 27.6 months and median of 5.5 months), relative to the participants in the Turkish (5-year median duration) and the Japanese study (mean ± SD 45.1 ± 73.2 months). The cross-cultural adaptation process of the FreNAQ-GR was performed according to international guidelines [31,32], reaching a consensus for a few minor issues arising during the forward and back translation stages. All committee members and pilot study participants rated the pre-final version of the scale as clearly phrased.

The total score was identical for the FreNAQ-GR and the FreNAQ-T (mean: 16.2), while it was less than half in the FreNAQ-J (mean ± SD: 7.7 ±5.4, and median: 6.0). Moreover, in the present study, no “floor” or “ceiling effects” were present for the total scale score, similar to both previous validation studies of the Fre-NAQ [30,33]. However, for individual scale items, only floor effects were present for five of the items (2, 3, 6, 7, and 8), with more than half of the participants (52.9%) selecting the lowest possible score for item 8. Interestingly, half of the participants (51.0%) rated item 9 with “often feels like that” (Table 3). For the FreNAQ-J [33], only floor effects were present, whereas the FreNAQ-T [30] registered both floor (in 6 of the items) and ceiling effects (in 5 of the items). The floor effect for item 8 of the FreNAQ-T [30] was particularly noticeable, as the majority of participants (86%) replied with ‘never feels like that’ (lowest possible score) to it. However, the total score of the questionnaire is always considered an indicator of the level of body awareness, and individual questions are not used separately. Differences in the duration of chronicity, pain, and disability levels, as well as cultural differences between populations, could have accounted for the differences in the scoring of the FreNAQ between the three studies.

The EFA conducted for this study revealed a 6-item single-factor structure of the FreNAQ-GR (Table 4), omitting items 7–9 that had low communalities. The previous two versions [30,33] confirmed the unidimensionality of the FreNAQ, however, with all items retained. No direct comparison can be made between the findings of the factor structure of the FreNAQ-GR and the previous two versions, as both previous studies had performed a Rasch analysis. However, a misfit in items 7, 8, and 9 of the FreNAQ-J was also noted [33]. The authors presented reasons for this misfit [33], suggesting that patients with NSCNP may have misinterpreted item 7 on the FreNAQ-J as acutely injured (thus swollen) rather than perceived as enlarged. Also, a possible contribution to the misfitting of item 7 (neck feels like it is enlarged (swollen)) and item 8 (neck feels like it has shrunk) was that these two items may be mutually exclusive (unless one’s impression of the size of their neck changes over time); therefore, participants would only endorse one of these two items.

The common variance explained by the FreNAQ-GR was 53.69% (>50%), considered acceptable for social research [47,49]. The factor that emerged consisted of six items with sufficiently strong loadings (0.63–0.71), all together solidly representing as a single factor [48,49], the concept of ‘neck awareness’ from the motor control, proprioception, and shape of the neck perception impairment point of view. It is intriguing that the items retained in this study were the respective first six items of the FreSHAQ-GR scale, also identified from EFA as representing a single factor [57]. Our work aligns with recent research, which suggests that a region-generic 6-item version of the Fremantle Body Awareness Questionnaire may uniformly examine the concept of ‘body awareness’ in a variety of musculoskeletal pathologies [58]. However, this generic version differs from the FreNAQ-GR and FreSHAQ-GR versions by one item (includes item 9 instead of item 2).

A correlational analysis was also used to examine the construct validity of the FreNAQ-GR, demonstrating highly significant correlations (*p* < 0.001) for both the 9- and 6-item versions of the FreNAQ-GR with the NDI (r = 0.33/0.29), the TSK (r = 0.46/0.41), and the PCS (r = 0.37/0.33). The FreNAQ-T significantly correlated with the NDI (r = 0.47), current pain intensity at rest (r = 0.40), at night (r = 0.32), and during activity (r = 0.31), the Beck Anxiety Inventory (r = 0.25), and several subscales of the SF-36 (r = 0.20–0.43); however, it marginally correlated with the TSK (r = 0.18) and with pain duration. Similarly, the FreNAQ-J significantly correlated with the NDI (r = 0.35), pain intensity with motion in the past 7 days (r = 0.36), the TSK (r = 0.28) and the PCS (r = 0.48); however, it did not correlate with pain intensity at rest (r = 0.23; *p* = 0.02). In all three studies, neck self-awareness was significantly associated both with patient disability and their cognitive domain affectivity. Indeed, pain-related fear of movement/re-injury, anxiety, and feelings of catastrophizing may distort body awareness and vice versa [30]. The FreNAQ-GR was not associated with the duration of symptoms or average pain intensity over the past week. In contrast, the two previous validations [30,33] took into account the VAS pain intensity felt under different situations (activity, rest, and at night), and significant associations were found in all of them except for pain at rest and the FreNAQ-J. Differences in VAS scale assessment conditions, as well as the different characteristics of the populations tested, may have accounted for the differences in the strength of the correlations between this and the two previous studies [30,33].

The FreNAQ-GR possessed good internal consistency for both the 9- and 6-item versions, respectively, as measured with the Cronbach’s α (0.80/0.826) and the McDonald’s ω (0.79/0.816) indices, with slightly higher values for the 6-item version for both indices. The internal consistency of the FreNAQ-J [33] was comparable (Chronbach’s α = 0.81) to the FreNAQ-GR, while for the FreNAQ-T [30], it was lower (Person Separation Index = 0.70), but also within the acceptable range.

The FreNAQ-GR demonstrated excellent test–retest reliability (n = 30, over a time interval of 5–7 days) for both the 9 and 6-item versions, with an ICC_2,1_(95% CI) = 0.98/0.98 (0.97–0.99/0.95–0.99), and low error values (SEM = 0.90/0.74 and MDC_95%_ = 2.49/2.05 points), respectively. Comparatively, the test–retest reliability of FreNAQ-J was very good, over a two-week time interval (n = 43), with an ICC_3,1_ (95% CI) = 0.81 (0.67 to 0.89) [33]. The test–retest reliability for the FreNAQ-T (n = 37) with a 3-day time interval was tested with differential item functioning (DIF) by time, with none of the items showing DIF, as well as an intraclass correlation coefficient of 0.711 reported [30]. None of the two previous studies reported the level of test–retest error. For this study, the MDC_95%_ was of very good level, with scores ±3 for the 9-item and ±2 for the 6-item FreNAQ-GR of a patient’s initial scores attributed to a true improvement or deterioration in patient condition and not the test–retest error.

This study confirmed the validity and reliability of the FreNAQ scale in relation to previous work [30,33]. A limitation present in this study was that, although this study confirmed the unidimensional structure of the FreNAQ-GR, it included 6 out of the 9 original scale items. The FreNAQ-J study also reported a similar issue for items 7–9, albeit with a different type of analysis. As a result, a confirmation of the FreNAQ-GR’s factor structure may be required in a subsequent study, although the number of participants used (n = 104) met the minimum criteria for EFA [47]. Additionally, a confirmatory factor analysis [58] for the FreSHAQ-GR should also be conducted. Furthermore, the FreNAQ-GR should be validated in patients with CNP of traumatic onset (i.e., whiplash), of various age groups, and occupational involvement. Regarding convergent validity, in the previous two validation studies of the FreNAQ [30,33] a weak but significant relationship of the FreNAQ with the pain intensity scales used was established. In a subsequent validation of the FreNAQ-GR, a possible differentiation of the pain intensity outcome under various movement (activity) and rest (daytime rest and at night) conditions might be required. The differences identified for the correlations between pain intensity and the FreNAQ could have resulted due to differences in patient pain characteristics and pain duration identified between studies. Finally, the responsiveness of the FreNAQ-GR scale should be tested in future investigations examining the effectiveness of movement-based [25,26] or body awareness-based [27,28] interventions.

## 5. Conclusions

The cross-cultural adaptation of the FreNAQ scale in Greek for patients with CNP was completed, presenting sufficient face and construct validity, supporting a 6-item version of the FreNAQ-GR. The results of the current study confirm that the scale possesses very good psychometric properties, comparable to the original scale, with good internal consistency and excellent test–retest reliability. Regarding its construct validity, the FreNAQ-GR correlated significantly with patient clinical status outcomes, and a factor analysis revealed a single-factor solution for the FreNAQ-GR.

Therefore, it is recommended for further use in the clinical and research environment involving Greek-speaking patients with neck pain conditions.

## Figures and Tables

**Table 1:** Descriptive statistics of the demographic characteristics (n = 104).

	Mean (SD)	Min-Max	Median (IQR)
Age (y)	44.0 (11.7)	25.0–70.0	44.0 (18.0)
Height (cm)	171 (8.7)	155.0–190.0	170.0 (11.0)
Body Mass (kg)	73.5 (14.4)	48.0–112.0	72.5 (24.0)
Body Mass Index (kg/m^2^)	25.1 (3.9)	18.1–35.9	24.4 (5.5)
Pain Duration (months)	17.4 (27.6)	3.0–144.0	5.5 (12.5)

y: years, cm: centimeters, kg: kilograms, m: meters

**Table 2:** Descriptive statistics of the questionnaires (n = 104).

	Mean (SD)	Min-Max	Median (IQR)
FreNAQ-GR	16.2 (5.9)	3.0–30.0	18.0 (8.0)
PI-VAS	4.0 (1.6)	0.0–8.0	4.0 (2.0)
NDI-GR	28.0 (9.6)	10.0–52.0	27.0 (14.0)
TSK-GR	42.6 (6.9)	26.0–59.0	43.0 (9.7)
PCS-GR	21.6 (8.1)	6.0–48.0	20.0 (9.7)

FreNAQ: Fremantle neck awareness questionnaire, NDI: neck disability index, PCS: pain catastrophizing scale, TSK: Tampa scale of kinesiophobia.

**Table 3:** Response percent frequencies per item and mean (SD) scores per item and total of the FreNAQ-GR.

Item	Never (%)	Rarely (%)	Occasionally (%)	Often (%)	Always (%)	Mean (SD)	Median (IQR)
1. My neck feels as though it is not part of the rest of my body	12.5	25	35.6	24.0	2.9	1.8 (1.0)	2.0 (2.0)
2. I need to focus all my attention on my neck to make it move the way I want it to	20.2	29.8	37.5	10.6	1.9	1.4 (1.0)	1.5 (1.0)
3. I feel as if my neck sometimes moves involuntarily, without my control	19.2	20.2	41.3	17.3	1.9	1.6 (1.0)	2.0 (1.0)
4. When performing everyday tasks, I do not know how much my neck is moving	7.7	18.3	40.4	26.9	6.7	2.1 (1.0)	2.0 (2.0)
5. When performing everyday tasks, I am not sure exactly what position my neck is in	6.7	16.3	45.2	25	6.7	2.1 (1.0)	2.0 (1.0)
6. I cannot perceive the exact outline of my neck	17.3	23.1	29.8	24	5.8	1.8 (1.2)	2.0 (2.0)
7. My neck feels like it is enlarged (swollen)	15.4	12.5	26	38.5	7.7	2.1 (1.2)	2.0 (2.0)
8. My neck feels like it has shrunk	52.9	23.1	11.5	0	3	0.8 (1.1)	0.0 (1.0)
9. My neck feels lopsided (asymmetrical)	9.6	6.7	19.2	51	13.5	2.5 (1.1)	3.0 (1.0)
Total Score						16.2 (5.9)	18.0 (8.0)

**Table 4:** Eigenvalues, communalities and factor loadings of exploratory factor analysis including the first 6 items of the FreNAQ-GR.

Factor	Initial Eigenvalues			Communalities	Factor Loadings
	Total	% of Variance	Cumulative %		
1	3.22	53.69	53.69	0.51	0.71
2	1.03	17.23	70.93	0.40	0.63
3	0.56	9.34	80.26	0.50	0.71
4	0.54	9.07	89.33	0.46	0.68
5	0.40	6.71	96.04	0.40	0.63
6	0.24	3.96	100.00	0.41	0.64

**Table 5:** Spearman’s correlation coefficients between the 9-item and 6-item FreNAQ-GR and NDI-GR, TSK-GR, and PCS-GR (n = 104).

	FreNAQ-GR (9-Item)	FreNAQ-GR (6-Item)
NDI-GR	0.33 **	0.29 **
TSK-GR	0.46 **	0.41 **
PCS-GR	0.37 **	0.33 **
FreNAQ-GR (9-item)		0.94 **

FreNAQ: Fremantle neck awareness questionnaire, NDI: neck disability index, PCS-GR: pain catastrophizing scale, TSK: Tampa scale of kinesiophobia, ** *p* < 0.001 (2-tailed).

**Table 6:** Descriptive statistics and reliability test–retest between-day reliability coefficients of the 9-item and 6-item FreNAQ-GR (n = 30).

9-Item	Day 1	Day 2	ICC_2,1_ (95% CI)	SEM	MDC_95%_
Mean (SD)	16.37 (5.07)	16.27 (5.03)	0.98 (0.97–0.99)	0.90	2.49
Median (IQR)	18.00 (4.25)	17.00 (5.25)			
Grand Mean = 16.32					
**6-Item**	**Day 1**	**Day 2**	**ICC_2,1_ (95% CI)**	**SEM**	**MDC_95%_**
Mean (SD)	10.70 (3.34)	10.63 (3.35)	0.98 (0.95–0.99)	0.74	2.05
Median (IQR)	11.00 (3.50)	11.00 (4.25)			
Grand Mean = 10.67					

## Data Availability

The data are available upon reasonable request.

## Supplementary Materials

The following supporting information can be downloaded at: https://www.mdpi.com/article/10.3390/healthcare12191985/s1, The Greek Version of the Fremantle Neck Awareness Questionnaire (FreNAQ-GR).
