# Supplementary material for: Cross-Cultural Adaptation, Reliability, and Validity of the Greek Version of the Fremantle Neck Awareness Questionnaire (FreNAQ-GR) in Patients with Chronic Neck Pain
Source: Healthcare (Basel). 2024 Oct 5;12(19):1985. doi: 10.3390/healthcare12191985 (PMC11477373; doi:10.3390/healthcare12191985)
Supplement: Supplementary file 1 [file healthcare-12-01985-s001.zip › healthcare-3230000-supplementary.pdf]

Ερωτηματολόγιο Σωματικής Αυτο-Αντίληψης του Αυχένα
The Greek Fremantle Neck Awareness Questionnaire (FreNAQ-GR)

Τα παρακάτω αποτελούν φράσεις που άλλοι ασθενείς μας ανέφεραν σε σχέση με το πως αισθάνονται τον επώδυνο αυχένα τους. Χρησιμοποιώντας την ακόλουθη κλίμακα, παρακαλούμε σημειώστε το βαθμό στον οποίο νιώθετε τον αυχένα σας κατά τον τρόπο που περιγράφεται παρακάτω **όταν είναι επώδυνος**.

0 = Ποτέ δεν τον αισθάνομαι έτσι
1 = Σπάνια τον αισθάνομαι έτσι
2 = Περιστασιακά / για κάποιο χρονικό διάστημα τον αισθάνομαι έτσι
3 = Συχνά / αρκετό χρονικό διάστημα τον αισθάνομαι έτσι
4 = Πάντα / το μεγαλύτερο χρονικό διάστημα τον αισθάνομαι έτσι

	**Ποτέ**	**Σπάνια**	**Περιστασιακά**	**Συχνά**	**Πάντα**
**1.** Νιώθω τον αυχένα μου σαν να μην είναι μέρος του υπόλοιπου σώματός μου					
**2.** Χρειάζεται να συγκεντρωθώ ολοκληρωτικά στον αυχένα μου έτσι ώστε να καταφέρω να τον κινήσω όπως θέλω					
**3.** Νιώθω τον αυχένα μου μερικές φορές σαν να κινείται ακούσια, χωρίς τον έλεγχο μου					
**4.** Όταν εκτελώ καθημερινές δραστηριότητες, δεν γνωρίζω ακριβώς πόσο κινείται ο αυχένας μου					
**5.** Όταν εκτελώ καθημερινές δραστηριότητες, δεν είμαι σίγουρος σε ποια ακριβώς θέση βρίσκεται ο αυχένας μου					
**6.** Δεν μπορώ να αντιληφθώ το ακριβές περίγραμμα του αυχένα μου					
**7.** Αισθάνομαι τον αυχένα μου μεγαλύτερο από το πραγματικό του μέγεθος (διογκωμένο)					
**8.** Αισθάνομαι τον αυχένα μου μικρότερο από το πραγματικό του μέγεθος (συρρικνωμένο)					
**9.** Αισθάνομαι τον αυχένα μου σε λοξή-μη συμμετρική θέση					

The Fremantle Neck Awareness Questionnaire (FreNAQ)

Here are some things which other patients have told us about how their painful neck feels to them. Using the following scale, please indicate the degree to which your neck feels this way **when you are experiencing neck pain**.

0 = Never feels like that
1 = Rarely feels like that
2 = Occasionally, or some of the time feels like that
3 = Often, or a moderate amount of time feels like that
4 = Always, or most of the time feels like that

	**Never**	**Rarely**	**Occasionally**	**Often**	**Always**
**1.** My neck feels as though it is not part of the rest of my body					
**2.** I need to focus all my attention on my neck to make it move the way I want it to					
**3.** I feel as if my neck sometimes moves involuntarily, without my control					
**4.** When performing everyday tasks I don't know how much my neck is moving					
**5.** When performing everyday tasks I am not sure exactly what position my neck is in					
**6.** I can't perceive the exact outline of my neck					
**7.** My neck feels like it is enlarged (swollen)					
**8.** My neck feels like it has shrunk					
**9.** My neck feels lopsided (asymmetrical)					
